# Changes to vertebrate tissue stable isotope (δ^15^N) composition during decomposition

**DOI:** 10.1038/s41598-019-46368-5

**Published:** 2019-07-09

**Authors:** Sarah W. Keenan, Jennifer M. DeBruyn

**Affiliations:** 1 University of Tennessee, Department of Biosystems Engineering and Soil Science, 2506 E.J. Chapman Drive, Knoxville, TN 37996 USA; 2 Present Address: South Dakota School of Mines & Technology, Department of Geology and Geological Engineering, 501 East St. Joseph Street, Rapid City, SD 57701 USA

**Keywords:** Stable isotope analysis, Environmental sciences

## Abstract

During carcass decomposition, tissues undergo biochemical changes: Cells autolyze, enteric microbes ferment cellular products, and tissues degrade. Ultimately, decomposition fluids are released as an ephemeral nitrogen (N) and carbon source to the surrounding environment. However, decomposition fluids are δ^15^N-enriched relative to body tissues, leading to a disconnect between starting tissue composition and ending fluid composition. It remains largely unknown when or if tissues exhibit δ^15^N enrichment postmortem despite the importance of tissue stable isotopes to ecologists. To test our hypothesis that tissues would become progressively δ^15^N-enriched during decay, soft tissues and bone were collected from beaver carcasses at five time points. All soft tissues, including muscle, were significantly δ^15^N-enriched compared to fresh tissues, but were not as enriched as decomposition fluids. Tissue breakdown is initially dominated by anaerobic autolysis and later by microbe and insect infiltration, and partly explains decay fluid isotopic enrichment. We speculate that after rupture, preferential volatilization of δ^15^N-depleted compounds (especially ammonia) contributes to further enrichment. These results constrain the timing, rate, and potential mechanisms driving carcass isotopic enrichment during decay, and suggest that found carcasses (e.g., road kill) should be used with caution for inferring trophic ecology as decay can result in significant postmortem δ^15^N enrichment.

## Introduction

Vertebrate tissue isotopic composition is widely used by ecologists to address questions related to broader ecosystem functioning, including population dynamics, animal migrations or ranges, provenance, age, and diet, which can be used to infer trophic-level interactions^1–5^. For animals that are difficult to observe directly in nature or for archaeological communities, these types of chemical fingerprints are invaluable for understanding ecosystems^6,7^. As a general rule, a 2 to 4‰ enrichment in tissue nitrogen (N) stable isotopic composition (δ^15^N) above the diet value is expected, resulting in increasing δ^15^N with increasing trophic level^8–10^. Under nutritional stress and/or shifts in the animal’s nitrogen balance, nitrogen enrichment patterns begin to deviate^11,12^, however this is usually only observed under the most extreme conditions (i.e., fasting, suboptimal health)^13,14^. Anabolic states, where protein synthesis increases (e.g. during pregnancy), leads to an overall decrease in hair δ^15^N values^15^, while catabolic states or starvation and high protein diets lead to δ^15^N-enrichment^11,16^.

Despite the potential for N balance and nutritional stress to alter tissue δ^15^N composition, stable isotopes still provide an important tool for ecological research. Prior studies evaluating animal ecology using stable isotopes have relied on the use of three broad types of samples: (1) Primary samples (i.e., muscle, hair, feces) collected from live animals or animals with a known time of death; (2) Found samples (i.e., salvaged road kill, scat, hair or feathers) with an unknown duration of exposure or postmortem interval; and (3) Previously collected and archived samples (i.e., bones and hair in lab or museum collections). Each sample type—primary, found, or archived—provides critical information about ecological interactions. Stable isotope analyses from primary samples from living animals, particularly those that are threatened or endangered, provide critical insights into habitat use, diet, and niche partitioning between species. For example, fur stable isotopic composition of wild-captured mouse lemurs revealed geographical differences in diet^2^. For animals that are difficult to track or with low population densities, found samples are the only viable materials available. For example, the coyote diet was reconstructed using stable isotopic composition of road kill and scat^5^. The use of archived specimens, often collected over decades and potentially from difficult to access localities or from endangered species, may represent the only viable sample source for reconstructing past environments. For example, museum specimens (macaque hair and bone) were used to infer distinct dietary (vegetation) and soil δ^15^N composition^3^.

All three sample types have potential limitations, including an important and often overlooked aspect of vertebrate tissues: postmortem decomposition. After death, an animal carcass goes through several decay stages, each characterized by chemical and physical changes to the tissues and organs, and results in the recycling and subsequent release of nutrients to the surrounding environment^17–19^. Changes to a carcass are driven by biological (particularly microbial) and autolytic processes^20–22^. The rapid pulse of nutrients, including carbon (C) and nitrogen (N), to the environment forms localized and ephemeral hotspots that stimulate micro-and macro-fauna and perturb biogeochemical cycling^23,24^. During a prior study of soil stable isotopic composition during decay, soils beneath decomposing North American beavers (*Castor canadensis*) rapidly increased from background δ^15^N values of 1 to 2‰ up to maximal values of ~12‰ during advanced decomposition^25^. A similar study of soil beneath decaying salmon in the Pacific Northwest observed a ~6.8‰ isotopic enrichment during decay^26^. However, the processes leading to such soil enrichment are not clearly understood; based on beaver bone collagen stable isotopic composition^27^, fluids released from carcasses were expected to be 2 to 4‰ enriched. However, decomposition fluid δ^15^N values from beaver carcasses ranged from 8 to 10‰^25^, suggesting that at some point during decay, beaver tissues/fluids become significantly isotopically-enriched. This was also observed with decaying salmon, where decomposition fluids were 1.5‰ enriched above fresh tissue^26^.

Tissue decomposition stable isotopic composition has only been studied in select aquatic taxa in laboratory incubations. Dolphin and sea turtle decomposition over 62 days revealed no significant changes to muscle and skin δ^15^N composition, which would suggest that fluids (based on prior studies^25,26^) rather than tissues become enriched during decay^28^. In contrast, muscle tissue samples from several marine vertebrates exhibited some ^15^N-enrichment after 8 days in the lab, with significant (up to 2.2‰) enrichment after 256 days^29^. On shorter timescales (5 days), fish muscle increased by 1.3‰ when allowed to air dry^30^, and after three days at > 20 °C, whale skin exhibited 6.4‰ enrichment^31^. While these controlled laboratory studies on tissue subsamples provide a critical first look at vertebrate tissue decomposition, it is unclear how applicable these results are to whole carcasses in natural ecosystems. In addition, these studies have been limited to muscle tissues, so it remains unknown how other tissues, such as the liver, lungs, heart, or intestinal tract, are altered during decay. As most animals die and decay in natural ecosystems, and decomposition processes involve multiple tissues simultaneously, observing decay of whole carcasses in nature is critical for better understanding decomposition. These observations are essential for assessing the suitability of naturally-decomposed tissues for ecology and forensics-based studies.

Given prior observations of carcass-derived fluid isotopic enrichment and mixed results with respect to muscle tissue isotopic changes in the previous lab-based studies, the goal of this study was to determine if and when tissues undergo stable isotopic enrichment during natural decomposition of whole animal carcasses. Analyses of vertebrate (North American beaver) tissue stable isotopic composition included: (1) characterizing the isotopic composition of a variety of tissues in fresh carcasses, establishing initial intra- and interindividual isotopic values; and (2) monitoring the isotopic composition of tissues throughout bloat and active decay to determine which tissues undergo isotopic enrichment prior to complete tissue breakdown. Soft tissues (i.e. muscle and liver) collected during active decay were expected to be ^15^N-enriched compared to fresh tissues. The more recalcitrant components of the animal, namely bones and hair, were not expected to undergo postmortem isotopic enrichment during this time frame.

## Materials and Methods

Salvaged nuisance North American beavers (*Castor canadensis*) were captured using Conibear traps, frozen at −20 °C within 24 hours, and stored frozen prior to beginning the experiment. Beavers were collected from various locations within an 8-month time period (August to April) in East Tennessee near Oak Ridge and Kingston and were provided by the US Department of Agriculture and Tennessee Wildlife Resources Agency. Animals weighed between 15 and 25 kg and all hides were intact. Beavers are generalist herbivores that feed on plant shoots, bark, and stems as well as other terrestrial and aquatic vegetation. Experiments were conducted at the University of Tennessee Forest Resources Research and Education Center at the Oak Ridge Forest. Because all animals used in this study were salvaged and not sacrificed for this project, no IACUC approval was required.

**Figure 1:**
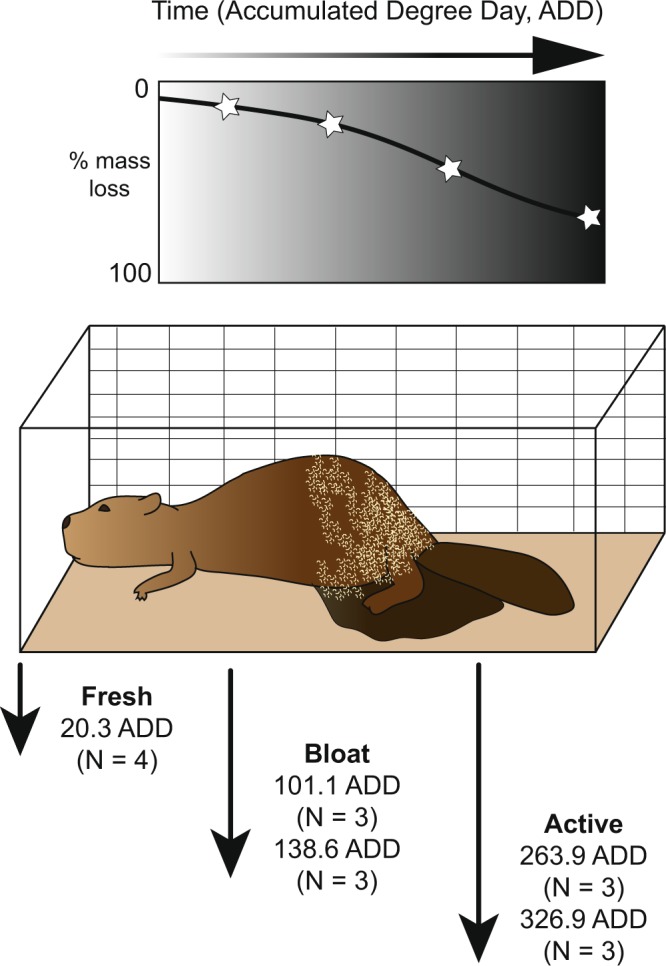
Schematic showing sampling timeline for beaver tissues during decay. At each sampling time, presented as accumulated degree days (ADD), tissues were collected from multiple individuals (N) and from a range of tissue types. Stars along the mass loss curve represent approximate sampling times along the decay trajectory.

Carcasses were placed in direct contact with the ground surface in wire scavenger prevention enclosures (1.19 × 0.74 × 0.81 m) to allow for natural decomposition to occur without vertebrate scavenging (in East Tennessee, scavengers were predominantly raccoons and vultures). Several stages during decomposition were targeted for tissue collection, including fresh (maximum of 24 to 36 hours postmortem prior to complete thawing), bloat (start of putrefaction and prior to rupture), and active (initial fluid release post-rupture and after extensive blowfly larvae colonization)^17,32^ (Fig. 1). Because vertebrate decomposition occurs in a continuum, beavers were destructively sampled at two times during bloat and two times during active decay to capture the full variability present within those decay stages. Beavers were allowed to thaw overnight before collecting fresh tissue samples, and carcass internal organs were still largely frozen during sampling.

**Figure 2:**
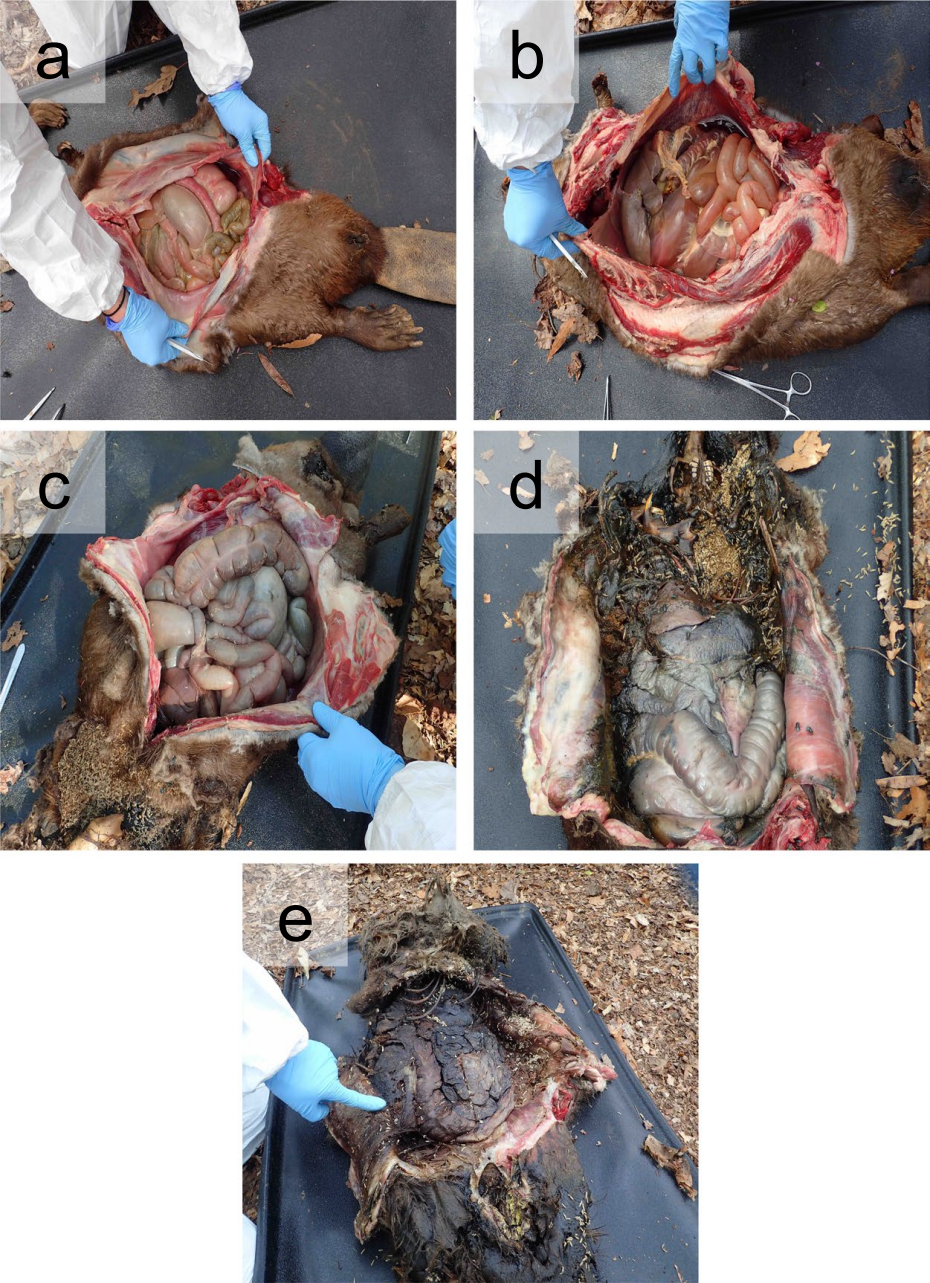
Photos of beaver internal organs prior to sampling at specific decay stages. (**a**) Carcasses reached early bloat at 101.1 ADD. (**b**) Late bloat (138.6 ADD) marked continued enlargement of the carcasses, visible accumulation of gasses in the GI tract, with some external blowfly larvae colonization. (**c**) Early active decay (263.9 ADD) was defined as the period after bloat when blowfly larvae had colonized the carcasses and the start of fluid release. (**d**,**e)** Late active decay (326.9 ADD) was the last point in decomposition where tissues were still identifiable and retained some structure. Blowfly larvae colonization was extensive and extended into the body cavity and most internal organs.

Due to the invasive nature of sampling internal organs, the carcasses were destructively sampled: 16 beavers were placed initially; four beavers were collected for the fresh time point, then three beavers were samples for each subsequent time point (Fig. 1). Fluids began to accumulate during the bloat stage and surrounded the organs (i.e., within the body cavity) (Fig. 2). Tissues in contact with decomposition fluids were not rinsed prior to collection because this is part of the natural decay process. Tissues remained identifiable throughout decomposition, except for the heart and lungs, which had completely degraded by the final active decay sampling time point and thus could not be sampled.

Tissues sampled from all time points included: from the left hindlimb—hair (short and long guard hairs), muscle, subcutaneous fat, cross section from the midshaft of the tibia; from the internal organs—liver, heart, right lung, small intestine, and hindgut contents. Only hair samples without contaminants (i.e., decomposition fluids, soil/sediment) were collected. To minimize contamination from fluids, subcutaneous fat, muscle, hair, and bone samples were collected first. Liver, heart, and lungs were collected prior to disrupting the gastrointestinal tract and collecting tissue from the small intestine, stomach contents, and hindgut. When present, blowfly larvae (maggots) were physically removed from tissues and were also collected for analyses.

Tissues and blowfly larvae were sealed in sterile sampling bags (Whirl-Pak, Nasco), flash-frozen in liquid nitrogen, and stored at −80 °C until freeze-drying. Samples were freeze-dried (Labconco, FreeZone) and stored at −20 °C until powdered for isotopic analysis. Tissues, except for hair, were homogenized by hand in a mortar and pestle, which was cleaned between use with methanol. Hair (both short and long guard hairs) was cut into fragments using methanol-sterilized scissors. Fragments from the base, mid-length, and tip of both hair types was combined for analysis to generate an averaged composition. For bones, any adhering soft tissue or periosteum was removed before powering in a mortar and pestle. As a cross section of bone was used, isotopic values of bone samples reflect total bone carbon (i.e. collagen-carbon and bioapatite carbonate-carbon). Because the goal of this study was to identify if tissues exhibit stable isotopic changes during decay, which may be partly due to changes to the lipid fraction, no tissues were chemically treated prior to freeze-drying and analyses. This includes retaining all lipids within samples, unlike some prior studies, which removed lipids prior to analyses^5,33^.

To allow for comparison with other studies utilizing animal hair, we cleaned two subsamples of hair using a 2:1 mixture of chloroform:methanol^6,34^. There was no difference in isotopic composition between cleaned and non-cleaned samples, therefore these samples were included as duplicate measurements.

Homogenized sample aliquots were transported to Washington University in St. Louis for stable isotopic analyses. Tissues and hair were weighed into 5 × 9 mm tin capsules (Costech Analytical Technologies Inc.) with sample mass optimized for tissue type and for carbon (C) and/or nitrogen (N) analyses (Supplementary Table S1). Fat and hindgut contents were analyzed for C and N separately due to the different masses required for each analysis. All other tissues were analyzed using a single sample. Both C and N are relevant parameters and were analyzed because previous studies observed changes C and N^29^, while others only observed changes to N^30,31^. Samples, standards, and blanks were loaded into a Costech Zero Blank autosampler and combusted in a Flash 2000 elemental analyzer. Tissue δ^13^C and δ^15^N values were measured on a Delta V Plus continuous-flow (Conflo IV), isotope-ratio-mass spectrometer. Standards included millet, acetanilide, and protein. Protein was used to evaluate linearity. Sample carbon and nitrogen isotopic values were corrected for sample size and instrument drift using protein, urea, and acetanilide. Analytic precision was < 0.2‰ for both carbon and nitrogen. Results are presented in δ notation as parts per mil (‰) where δ^13^C = [((^13^C/^12^C_sample_/^13^C/^12^C_standard_) − 1) × 1,000] and δ^15^N = [((^15^N/^14^N_sample_/^15^N/^14^N_standard_) − 1) × 1,000]. Vienna Pee Dee Belemnite was used as the carbon standard and air was used as the nitrogen standard.

### Data analysis and statistics

Four animals were sampled immediately after thawing and represent the fresh beaver stable isotopic composition. Data obtained from the four ‘fresh’ individuals were combined and averaged for statistical analyses. To capture the dynamic changes that occur to tissues during decay, four time points were selected for sampling: early bloat, late bloat, early active decay, late active decay. At each of these time points, 3 beavers were destructively sampled and analyzed; mean and standard deviation of N = 3 beavers were calculated. Combined with the initial samples, this gave a total of five time points. For analyses of changes between decay stage, the two sampling times within each decay stage (bloat or active decay) were combined for data analysis, yield three stages for comparison: fresh, bloat, and active. One-way ANOVAs (p < 0.05) were conducted using SigmaPlot (version 14.0) with Holm-Sidak post-hoc testing to test for significant differences between tissue types for each of the response variables. Paired t-tests were used to identify if decay stages (bloat vs. active) differed significantly (Welch t-test, R)^35^.

Linear regression analysis was used to assess relationship between stable N isotopic fractionation and time. Tissue sampling times were calculated as accumulated degree days (ADD)^36^, based on temperatures obtained from a NOAA weather station located at the Oak Ridge Forest site (GHCND:USW00003841). ADD is calculated as the sum of daily average temperatures (°C), using the observed minimum and maximum, from placement until sampling. ADD is a more appropriate metric for decomposition rather than time because it accounts for temperature, which can enhance or restrict biological reaction rates, particularly bacterial and blowfly larvae growth^36^.

Animals typically exhibit 2 to 4‰ enrichment in tissue stable δ^15^N composition above their diet^8,10^. To evaluate whether decay alters the expected diet-driven fractionation, Δ^15^N_animal-diet_ was calculated using the average δ^15^N value of gut contents (diet) from the four fresh beavers (mean ± s.d., 0.55 ± 2.1‰; range of −1.49 to 3.21‰) as the predicted starting diet composition.

## Results

### Fresh beaver tissue isotopic composition

**Figure 3:**
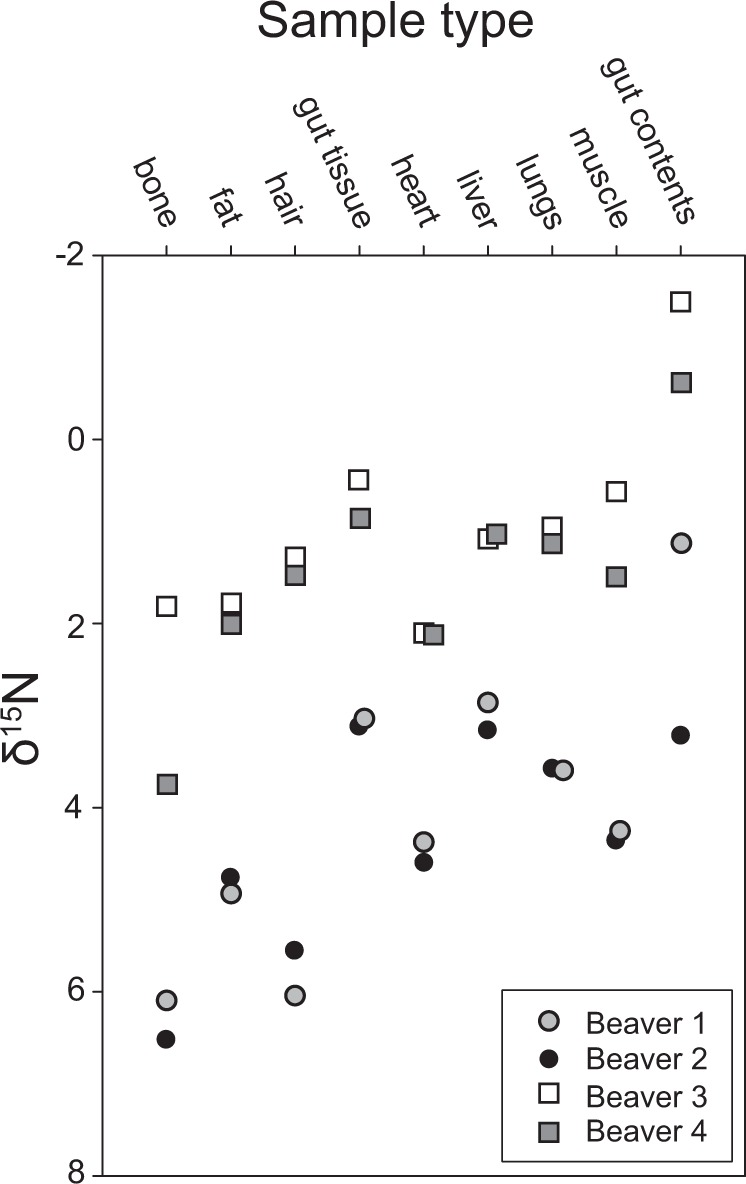
Stable δ^15^N isotopic composition of fresh beaver soft tissues, bone, hair, and gut contents (N = 4).

Gut contents were isotopically-depleted compared to soft tissues and bone from the same animal (Fig. 3), and mean tissue δ^15^N enrichment above gut contents ranged from 1.3 to 4.1‰. Tissue stable isotopic composition of one individual (Fig. 3, Beaver 2) deviated from typical enrichment, with abnormally enriched gut contents compared to the three other individuals and relative to other tissue stable isotopic compositions. Mean (± s.d.) soft tissue δ^15^N composition ranged from 1.9 ± 1.4‰ for gut tissue to 3.4 ± 1.7‰ for fat, while bone and hair were slightly more enriched and exhibited greater variation (Table 1). The Δ^15^N_animal-diet_ values for fresh tissues ranged from 1.3 ± 1.4‰ for gut tissue up to 4.0 ± 2.2‰ in bone and all samples exhibited high variability, up to ±2.6‰ in hair samples. The C/N ratio of fresh tissues ranged from 49.2 ± 36.3 for fat to 3.9 ± 0.6 for all other soft tissues, bone, and hair (Table 1; Supplementary Table S2).

**Table 1:** C and N composition of beaver tissues as a function of decay stage.

Decay Stage	Tissue type	δ^15^N (‰)		Wt. %N		δ^13^C (‰)	Wt. %C		C:N		−Ln(%N)		−Ln(%C)	Δ^15^N_animal-diet_	
Fresh	Bone	4.5 ± 2.2		3.6 ± 0.1		−24.7 ± 1.1	13.7 ± 1.8		3.8 ± 0.4		−1.3 ± 0.0		−2.6 ± 0.1	4.0 ± 2.2	
Bloat	Bone	4.4 ± 0.6		3.8 ± 0.2		−23.1 ± 1.9	13.7 ± 1.1		3.6 ± 0.3		−1.3 ± 0.1		−2.6 ± 0.1	3.8 ± 0.6	
Active	Bone	5.9 ± 1.3		4.0 ± 0.6		−21.7 ± 1.9	14.0 ± 2.2		3.5 ± 0.1		−1.4 ± 0.1		−2.6 ± 0.1	5.3 ± 1.3	
Fresh	Fat	3.4 ± 1.7	ab	0.7 ± 0.1	ab	−34.3 ± 1.0	33.1 ± 26.0		49.2 ± 36.3		0.4 ± 0.2		−3.3 ± 0.6	2.8 ± 1.7	a
Bloat	Fat	3.5 ± 0.9	a	0.7 ± 0.2	a	−33.3 ± 1.7	33.8 ± 22.2		54.7 ± 38.0		0.4 ± 0.4		−3.4 ± 0.6	2.9 ± 0.9	a
Active	Fat	5.8 ± 1.3	b	1.2 ± 0.5	b	−32.0 ± 1.6	19.6 ± 0.3		18.7 ± 6.4		−0.1 ± 0.4		−3.0 ± 0.0	5.3 ± 1.3	b
Fresh	Hair	3.6 ± 2.6		13.1 ± 0.5		−25.2 ± 0.4	41.6 ± 1.1		3.2 ± 0.1		−2.6 ± 0.0		−3.7 ± 0.0	3.0 ± 2.6	
Bloat	Hair	4.4 ± 0.5		13.8 ± 0.8		−24.1 ± 2.2	43.3 ± 1.9		3.1 ± 0.1		−2.6 ± 0.1		−3.8 ± 0.0	3.8 ± 0.5	
Active	Hair	5.8 ± 0.2		13.5 ± 1.3		−23.3 ± 0.9	44.1 ± 1.1		3.3 ± 0.4		−2.6 ± 0.1		−3.8 ± 0.0	5.2 ± 0.2	
Fresh	Gut contents	0.6 ± 2.1	a	2.0 ± 0.6		−30.0 ± 1.4	40.1 ± 3.4		22.2 ± 9.1		−0.6 ± 0.3		−3.7 ± 0.1		
Bloat	Gut contents	2.8 ± 1.4	ab	2.9 ± 0.7		−29.4 ± 1.6	40.6 ± 1.6		15.3 ± 6.1		−1.0 ± 0.3		−3.7 ± 0.0	2.3 ± 1.4	
Active	Gut contents	5.0 ± 2.6	b	2.7 ± 1.0		−28.8 ± 0.9	41.2 ± 1.3		16.8 ± 10.1		−0.9 ± 0.4		−3.7 ± 0.0	4.5 ± 2.6	
Fresh	Gut tissue	1.9 ± 1.4	a	9.0 ± 1.0		−27.5 ± 1.0	42.5 ± 2.9	a	4.8 ± 0.8	a	−2.1 ± 0.1		−3.7 ± 0.1	1.3 ± 1.4	a
Bloat	Gut tissue	3.4 ± 0.9	ab	10.0 ± 1.3		−26.9 ± 1.5	45.1 ± 3.3	ab	4.5 ± 0.4	b	−2.3 ± 0.1		−3.8 ± 0.1	2.9 ± 0.9	ab
Active	Gut tissue	5.1 ± 1.4	b	8.2 ± 1.5		−27.6 ± 0.9	48.6 ± 2.3	b	6.1 ± 1.0	b	−2.1 ± 0.2		−3.9 ± 0.0	4.6 ± 1.4	b
Fresh	Heart	3.3 ± 1.4	a	11.4 ± 1.4		−27.2 ± 1.0	46.1 ± 3.0		4.1 ± 0.4		−2.4 ± 0.1		−3.8 ± 0.1	2.7 ± 1.4	a
Bloat	Heart	4.6 ± 0.8	ab	12.4 ± 0.3		−26.3 ± 1.6	48.2 ± 1.1		3.9 ± 0.2		−2.5 ± 0.0		−3.9 ± 0.0	4.0 ± 0.8	ab
Active	Heart	6.2 ± 1.4	b	11.9 ± 0.8		−25.5 ± 1.2	48.2 ± 1.3		4.1 ± 0.2		−2.5 ± 0.1		−3.9 ± 0.0	5.7 ± 1.4	b
Fresh	Liver	2.0 ± 1.1	a	10.2 ± 0.8		−28.2 ± 1.1	48.1 ± 2.6	a	4.7 ± 0.2		−2.3 ± 0.1		−3.9 ± 0.1	1.5 ± 1.1	a
Bloat	Liver	3.7 ± 1.2	a	9.6 ± 1.7		−28.5 ± 1.9	51.6 ± 2.7	ab	5.6 ± 1.4		−2.2 ± 0.2		−3.9 ± 0.1	3.2 ± 1.2	ab
Active	Liver	5.4 ± 1.1	b	8.4 ± 1.2		−28.2 ± 1.7	54.9 ± 2.9	b	6.6 ± 1.0		−2.1 ± 0.1		−4.0 ± 0.1	4.8 ± 1.1	b
Fresh	Lungs	2.3 ± 1.5	a	12.4 ± 0.5	a	−26.6 ± 1.0	45.5 ± 2.6		3.7 ± 0.2	a	−2.5 ± 0.0	a	−3.8 ± 0.1	1.8 ± 1.5	a
Bloat	Lungs	3.7 ± 0.8	ab	12.8 ± 1.0	b	−26.1 ± 1.5	46.8 ± 2.3		3.7 ± 0.3	b	−2.5 ± 0.1	b	−3.8 ± 0.0	3.1 ± 0.8	ab
Active	Lungs	5.3 ± 1.0	b	9.7 ± 0.6	b	−26.3 ± 2.1	48.3 ± 2.1		5.0 ± 0.1	b	−2.3 ± 0.1	b	−3.9 ± 0.0	4.7 ± 1.0	b
Fresh	Muscle	2.7 ± 1.9	a	13.2 ± 1.2		−25.9 ± 0.8	43.8 ± 0.8	a	3.3 ± 0.3	a	−2.6 ± 0.1		−3.8 ± 0.0	2.1 ± 1.9	a
Bloat	Muscle	4.1 ± 0.6	ab	13.3 ± 0.6		−25.4 ± 1.7	45.4 ± 0.8	b	3.4 ± 0.1	ab	−2.6 ± 0.0		−3.8 ± 0.0	3.6 ± 0.6	ab
Active	Muscle	5.7 ± 0.8	b	12.9 ± 0.4		−25.0 ± 1.2	46.7 ± 0.3	c	3.6 ± 0.1	b	−2.6 ± 0.0		−3.8 ± 0.0	5.2 ± 0.8	b
Bloat	Blowfly larvae	6.9		9.6		−25.7	42.8		4.5		−2.3		−3.8	6.4	
Active	Blowfly larvae	7.4 ± 0.5		8.2 ± 1.0		−26.5 ± 1.4	48.0 ± 3.0		5.9 ± 1.0		−2.1 ± 0.1		−3.9 ± 0.1	6.8 ± 0.5	

Data are presented as mean ± standard deviation. Significant differences between decay stages within each tissue type as determined using a one-way ANOVA indicated by different letters. The negative natural log of the %N and %C are also included and are abbreviated as -Ln(%N) and -Ln(%C). Full dataset available in Supplementary Table S2.

### Decomposing beaver tissue physical changes and isotopic composition

Beaver tissues underwent visible physical changes during decomposition (Fig. 2). The transition from early to late bloat (101.1 to 138.6 ADD) included enhanced gas buildup within the gastrointestinal tract, particularly the small intestines (Fig. 2b). At both bloat sampling stages, fluid began to pool within the body cavity. Animals sampled during early active decay (263.9 ADD) exhibited some fluid release and mass loss, blowfly larvae colonization of some limbs and the face/skull (Fig. 2c). At this stage, blowfly larvae had entered the body cavity and several were observed within heart tissue and the stomach of one animal. Blowfly larvae had also penetrated into the hindlimb muscles of one beaver, and the subcutaneous fat within this area contained pockets or bubbles. Internal organs began to discolor, notably the gastrointestinal tract, turning a light purple to grey color.

By late active decay, blowfly larvae penetrated into a large portion of the internal organs, completely degrading the heart in all beavers (326.9 ADD) (Fig. 2d). The lungs also degraded rapidly. Lung tissue was only recovered from two of the three animals sampled during early active decay, and lung tissue had completely degraded by late active decay in all three beavers (326.9 ADD). The internal organs in one of the animals sampled during late active decay were largely desiccated and the liver was almost completely consumed by blowfly larvae (Fig. 2e). Internal organs continued to discolor, turning a deep purple to almost black in some animals.

**Figure 4:**
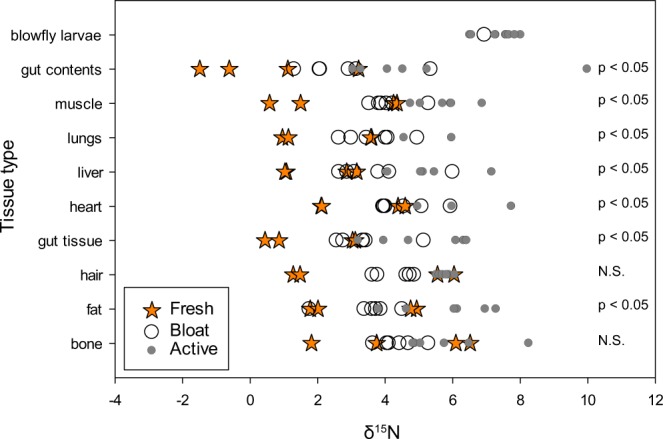
δ^15^N composition of beaver tissues during decomposition. All tissues, except for bones and hair exhibited significant increases as decomposition progressed (p-values and F-values presented in Supplementary Table S3 and refer to significant differences between fresh and active samples as determined by a t-test).

**Figure 5:**
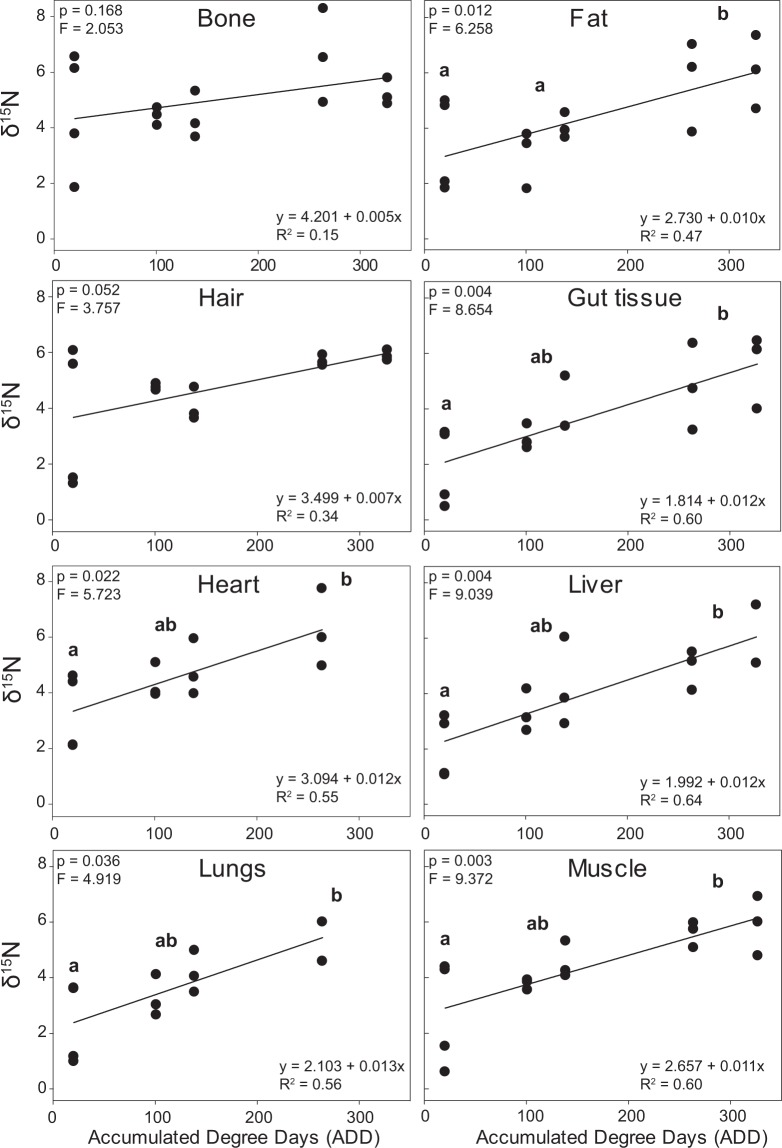
Changes to stable N isotopic fractionation over time. There is a significant linear increase in isotopic enrichment relative to diet during decay for all soft tissues and hair. The linear relationship between δ^15^N and ADD was not significant for bone. Heart and lung tissue were no longer identifiable by the final active sampling time point (326.9 ADD) and could not be sampled.

In addition to significant physical changes to tissues during decay, tissues also underwent chemical changes. Tissues began to exhibit some but not statistically significant isotopic enrichment during the bloat stage (101.1 to 138.6 ADD), with δ^15^N isotopic enrichment occurring to gut contents, gut tissue, heart, lungs, and muscle (Table 1). The greatest change in tissue δ^15^N composition as well as % C and % N occurred during active decomposition (263.9 to 326.9 ADD) (Figs. 4, 5, Table 1). All samples collected during active decomposition, except those obtained from bones and hair, exhibited significant ^15^N-enrichment compared to fresh tissues (Supplementary Table S3); there were no significant differences between fresh and bloat, and liver and fat were significantly different between bloat and active decay samples. Fat δ^15^N composition increased from 3.4 ± 1.7‰ in fresh beavers to 5.8 ± 1.3‰ in actively decomposing animals, a ~2‰ enrichment. Muscle, lungs, liver, heart, and gut tissue all exhibited ~3‰ enrichment in actively decaying tissues compared to fresh (Table 1). By the final sampling during active decay (326.9 ADD), Δ^15^N_animal-diet_ values were up to 7.2‰ (heart tissue; Table 1, Supplementary Table S2).

In contrast to significant changes to tissue stable δ^15^N composition during decay, there were no significant changes to δ^13^C within each tissue type (Supplementary Table S3). Fat samples were ^13^C-depleted compared to other tissues, ranging from −34.3 ± 1.0‰ in fresh animals to −32.0 ± 1.6‰ in actively decomposing beavers. Bones were comparatively enriched, ranging from −24.8 ± 1.1 to −21.7 ± 1.9‰ in fresh and actively decaying beavers, respectively (Table 1).

**Figure 6:**
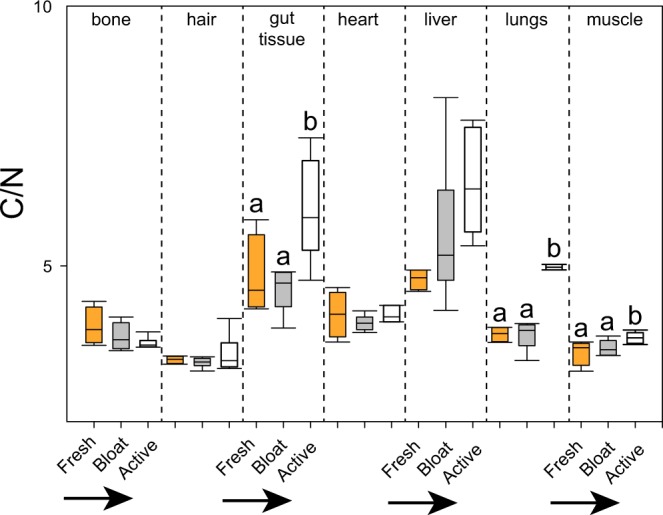
Changes to C/N ratio in tissues during decomposition. Fresh (orange), bloat (grey), and active tissue (white) tissue C/N values varied by tissue type as well as decay stage. Lowercase letters indicate significant differences between times within a tissue type (one-way ANOVA, Holm-Sidak post-hoc test).

The C/N ratios of the gut tissue, lungs, and muscle increased significantly during active decay (Fig. 6). Liver samples exhibited an overall trend of increasing C/N ratio during decay, however high variability between replicate carcasses during bloat resulted in an inability to detect significant differences. There were no significant changes observed in other tissues.

## Discussion

Fresh beaver tissue δ^15^N isotopic composition varied between individual carcasses, but was a mean of 2 to 6‰ enriched over diet (Fig. 4), slightly above the typical values in other vertebrates (2 to 4‰)^8–10^. Although the beavers were sourced from the same geographic region (an approximately 96 km^2^ area), there was variability in δ^15^N composition, with two of the four individuals exhibiting lower δ^15^N composition, and two exhibiting enriched δ^15^N values. These variations in fresh beaver tissue isotopic composition might reflect differences in foraging location, vegetation type consumed, and individual physiological variability^37,38^. Additionally, differences may also reflect seasonal variation in diet because animals may have been collected recently (i.e., summer) or may have been trapped in the winter where they may have experienced nutritional stress^12,38^. The sporadic nature of obtaining salvaged nuisance animals, which were immediately frozen and stored after collection, limits assigning a precise date or season of capture. In particular, the contents of the hindgut of beaver 2 were 2 to 6‰ enriched in δ^15^N compared to the gut contents other individuals, suggesting that this individual may have consumed an ^15^N-enriched food source prior to capture. Gut content δ^13^C isotopic composition ranged from −31.0 to −28.1‰ (mean −30.0 ± 1.4‰), which suggests beavers are consuming C3 plants with similar C stable isotopic compositions.

By the time carcasses reached early bloat (101.1 ADD), internal organs were visibly enlarged, particularly the small intestines (Fig. 2a), and enlargement and discoloration persisted through late bloat (138.6 ADD) (Fig. 2b). By active decay (263.9 to 326.9 ADD), carcasses underwent dramatic visible changes, culminating in almost complete desiccation in one of the animals sampled (Fig. 2e). The observed physical changes to beaver tissues were driven by changes to biological communities residing within and on the animals as well as insect scavenging. Insect activity (primarily flies) initiated within 24 hours of carcass placement. During decay, some tissue types were targeted by insect scavengers. There was noticeable blowfly larvae movement from the nose and mouth inwards starting during bloat, and extensive migration towards internal organs during early active decay. Heart tissue was the first internal organ visibly colonized by maggots during early active decay (263.9 ADD) and was completely degraded by late active decay (326.9 ADD). Muscle was also colonized by blowfly larvae during late active decay. Insects were able to migrate/burrow inwards from physical disruption (i.e., ruptures or tears) in the hide. Overall, by late active decay, blowfly larvae had invaded most tissues, including liver, stomach, and intestines. Lung tissues seemed to degrade before or near-synchronous with blowfly larvae invasion.

Despite inter-animal variability in fresh diet and fresh tissue δ^15^N isotopic composition, there was a clear trend of increasing ^15^N-enrichment over time during decomposition for all tissues except for bone and hair (Supplementary Table 3). Tissues that do show enrichment during decay were significantly enriched in δ^15^N in active decay samples compared to fresh tissues (Supplementary Table 2). In a prior study of several marine vertebrates, some ^15^N-enrichment was observed after 8 days in muscle tissue subsamples held in open and closed containers in the lab, with significant (up to 2.2‰) enrichment after 256 days^29^. Others observed significant enrichment in whale skin subsamples exposed > 20 °C for 3 days^31^. A study of whole animal degradation (fishes) also showed comparable significant ^15^N-enrichment^30^; however, the study was limited by conducting the decomposition experiments in a lab setting, removing natural colonization by insects observed in our study. These prior studies as well as the current study contrast with previous research of dolphin and turtle tissues where no significant enrichment was observed after 62 days^28^. In all prior studies, subsampling of the same tissue/carcass over time or removing tissue samples from the rest of the carcass combined with precluding natural decomposition processes (i.e., insect colonization) may not accurately represent processes experienced by whole carcass during decay in natural environments.

Tissue stable isotope enrichment observed in this study is likely due to a combination of processes and mechanisms, including tissue autolytic and biological breakdown, coupled with release of gas and/or fluids^21,22^. A range of gases are released during protein and amino acid breakdown associated with animal decay, including nitrogen compounds like cadaverine (NH_2_(CH_2_)_5_NH_2_) and putrescine (NH_2_(CH_2_)_4_NH_2_)^39,40^, as well as sulfur compounds, such as H_2_S^41^. N-containing gases, such as N_2_O, N_2_, and NH_3_, are likely ^14^N-enriched, leaving behind ^15^N-enriched residual N compounds and fluids. The release of nitrogen volatile organic compounds peaks during active decay^40^, which corresponds to the period of decay where most tissues exhibited significant δ^15^N isotopic enrichment. Animal tissues undergo isotopic fractionation during extreme nutritional stress or fasting^12^, and the processes occurring during catabolic conditions may also occur during cell death and breakdown. Tissue δ^13^C does not change in studies of animal tissues under nutritional stress, and we also did not see in our study a change in δ^13^C composition in decaying tissues. Other studies have found varying results with respect to δ^13^C composition^28–31^, and this may reflect differences in removal or loss of lipids prior to analyses. However, these previous studies show generally small changes in δ^13^C (as reviewed by Perkins *et al*.^30^). Given the small sample size here with large variation in δ^13^C and averaging values across individuals within a time-period, any small directional change in δ^13^C due to decomposition may have been masked by the analyses and is a limitation of our experimental design, which required destructive sampling. This may also explain no detectable changes for other tissues and response variables with large variance (such as bone and hair).

Fresh tissues exhibited a fractionation factor of 2 to 6‰. However, by active decay tissues exhibited at fractionation factor up to 7.2‰ (heart tissue; Supplementary Table S2). This emphasizes that by active decay, soft tissues no longer provide a reliable resource to reconstruct animal diet or trophic level, and for many tissues, including gut tissue, liver, heart, lungs, and muscle, the fractionation factor begins to exhibit further enrichment during bloat. Studies utilizing road kill or animal tissues obtained without a known time since death, particularly in warm and/or humid climates where decay rates are elevated^42^, should interpret trophic position and/or diet with caution.

Several tissues, including gut tissue, lungs, and muscle, exhibited a significant increase in C/N over time, similar to a previous study^30^. Changes to C/N were driven by a significant increase in wt. % C for gut tissue and muscle, while changes to C/N in the lungs were due to a significant decrease in wt. % N and a non-significant increase in wt. % C (Table 1). While no significant change was observed in liver C/N ratio, these tissues underwent a significant increase in wt. % C. There may be several processes occurring simultaneously to explain the observed shifts in wt. % C and N, and the resulting C/N ratios. First, anaerobic host-associated microorganisms increase during decay^21^, and are known to colonize tissues shortly after host death, preferentially invading tissues adjacent to the gastrointestinal tract^20,22^. Increased tissue wt. % C may reflect enhanced microbial colonization of tissues. Second, increased microbial biomass may potentially be coupled with a loss of N through tissue breakdown and volatile organic compound release driven by anaerobic heterotrophic microorganisms sourced from the gastrointestinal tract^39,40^.

**Figure 7:**
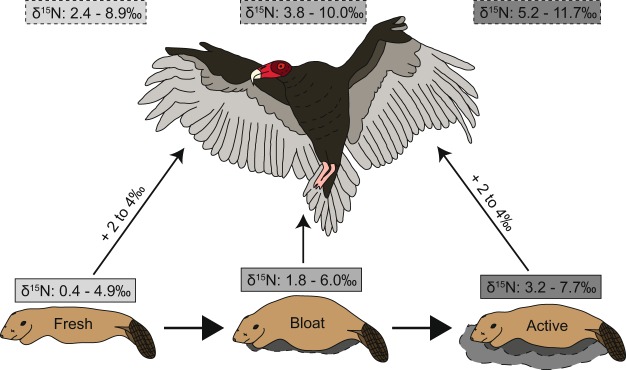
Schematic diagram emphasizing the potential trophic enrichment of scavengers feeding on fresh, bloated, or actively decaying tissues. Estimated resulting scavenger tissue is based on an enrichment factor of 2 to 4‰.

In addition to decomposition exerting a significant influence on carrion soft tissue δ^15^N stable isotopic composition, which can influence trophic level or dietary interpretations of the animal, these results have broader implications for other ecological studies. Vertebrates that consume carrion (i.e., vultures) feed on fresh to partially decayed carcasses^43^, and the further that tissues have progressed in decay, the greater the δ^15^N composition. Therefore, an animal scavenging bloated carrion will acquire different δ^15^N signatures (Fig. 7) than if it fed on fresh carrion, which may be misinterpreted as consuming a food source from a higher trophic position.

Postmortem alteration of vertebrate tissues has the potential to alter the original δ^15^N composition. For some sample types, such as fur and bone, which are routinely used to study animal diet, migration, and trophic ecology, postmortem isotopic enrichment was not observed to significantly change over time; however, there is the potential that collagen may undergo fractionation on longer timescales and this may be observed by increasing the sample size. Temperature is known to significantly influence rates of decomposition^42,44^, and tissues may undergo more pronounced fractionation during warmer times of the year compared to the fall or winter.

## Conclusions

Vertebrate tissues undergo significant physical and chemical transformations during decomposition. The changes to tissue stable isotopic composition documented in this study are an important first look at whole carcasses changes in a natural ecosystem. It should be noted that these data represent an end-member condition where vertebrate scavenging is minimized. In cases where scavengers consume soft tissues, exposing internal organs and tissues to oxygenated conditions and greater insect activity generally speeds up the rate of decomposition, and would likely affect the isotopic values. Changes to soft tissue isotopic composition are driven by both autolytic processes related to cell death and to biotic processes driven by micro- and macro-fauna. Given the large starting variability in δ^15^N for some tissues, particularly bone, there may have been changes that were not detected. But, any undetected changes would have been smaller than the range of δ^15^N values. We caution the use of vertebrate soft tissues for stable isotopic analyses if time since death is unknown as autolytic and biotic processes have the potential to lead to significant postmortem ^15^N-enrichment, and these changes are going to be climate and environment-specific. Subsequent research focusing on the physiochemical changes to animal tissues during decay and particularly the gases released during decay are warranted.

## Supplementary information

Supplementary Information

## Data Availability

All data generated or analyzed during this study are included in the published article and Supplementary Information.

## References

[1] Hobson, K. A. Isotopic tracking of migrant wildlife in *Stable isotopes in ecology and environmental science* (eds. Michener, R. & Lajtha, K.) 155–176 (Blackwell Publishing Ltd., 2007).

[2] Crowley BE (2011). Explaining geographical variation in the isotope composition of mouse lemurs (*Microcebus*). J. Biogeogr..

[3] O’Regan HJ (2008). Modern macaque dietary heterogeneity assessed using stable isotope analysis of hair and bone. J. Hum. Evol..

[4] Minagawa M, Wada E (1984). Stepwise enrichment of ^15^N along food chains: further evidence and the relation between δ^15^N and animal age. Geochim. Cosmochim. Acta.

[5] Reid REB, Koch PL (2017). Isotopic ecology of coyotes from scat and road kill carcasses: a complementary approach to feeding experiments. PLoS One.

[6] Hobson KA, McLellan BN, Woods JG (2000). Using stable carbon (δ^13^C) and nitrogen (δ^15^N) isotopes to infer trophic relationships among black and grizzly bears in the upper Columbia River basin, British Columbia. Can. J. Zool..

[7] Hedges REM, Reynard LM (2007). Nitrogen isotopes and the trophic level of humans in archaeology. J. Archaeol. Sci..

[8] Deniro MJ, Epstein S (1981). Influence of diet on the distribution of nitrogen isotopes in animals. Geochim. Cosmochim. Acta.

[9] Schoeninger MJ, DeNiro MJ (1984). Nitrogen and carbon isotopic composition of bone collagen from marine and terrestrial animals. Geochim. Cosmochim. Acta.

[10] Perkins MJ (2014). Application of nitrogen and carbon stable isotopes (δ^15^N and δ^13^C) to quantify food chain length and trophic structure. PLoS One.

[11] Gannes LZ, OBrien DM, Del Rio CM (1997). Stable isotopes in animal ecology: assumptions, caveats, and a call for more laboratory experiments. Ecology.

[12] Hobson KA, Alisauskas RT, Clark RG (1993). Stable-nitrogen isotope enrichment in avian tissues due to fasting and nutritional stress: implications for isotopic analyses of diet. Condor.

[13] Kempster B (2007). Do stable isotopes reflect nutritional stress? Results from a laboratory experiment on song sparrows. Oecologia.

[14] Loudon JE, Sponheimer M, Sauther ML, Cuozzo FP (2007). Intraspecific variation in hair δ^13^C and δ^15^N values of ring-tailed lemurs (*Lemur catta*) with known individual histories, behavior, and feeding ecology. Am. J. Phys. Anthropol..

[15] Fuller BT (2004). Nitrogen balance and δ^15^N: why you’re not what you eat during pregnancy. Rapid Commun. Mass Sp..

[16] Fuller BT (2005). Nitrogen balance and δ^15^N: why you’re not what you eat during nutritional stress. Rapid Commun. Mass Sp..

[17] Payne JA (1965). A summer carrion study of the baby pig *Sus scrofa* Linnaeus. Ecology.

[18] Payne JA, King EW, Beinhart G (1968). Arthropod succession and decomposition of buried pigs. Nature.

[19] Vass AA, Bass WM, Wolt JD, Foss JE, Ammons JT (1992). Time since death determinations of human cadavers using soil solution. J. Forensic Sci..

[20] Javan GT, Finley SJ, Abidin Z, Mulle JG (2016). The thanatomicrobiome: a missing piece of the microbial puzzle of death. Front. Microbiol..

[21] Hyde ER, Haarmann DP, Lynne AM, Bucheli SR, Petrosino JF (2013). The living dead: bacterial community structure of a cadaver at the onset and end of the bloat stage of decomposition. PLoS One.

[22] Tuomisto S, Karhunen PJ, Vuento R, Aittoniemi J, Pessi T (2013). Evaluation of postmortem bacterial migration using culturing and real-time quantitative PCR. J. Forensic Sci..

[23] Aitkenhead-Peterson JA, Owings CG, Alexander MB, Larison N, Bytheway JA (2012). Mapping the lateral extent of human cadaver decomposition with soil chemistry. Forensic Sci. Int..

[24] Towne EG (2000). Prairie vegetation and soil nutrient responses to ungulate carcasses. Oecologia.

[25] Keenan SW, Schaeffer SM, Jin VL, DeBruyn JM (2018). Mortality hotspots: nitrogen cycling in forest soils during vertebrate decomposition. Soil Biol. Biochem..

[26] Wheeler TA, Kavanagh KL (2017). Soil biogeochemical responses to the deposition of anadromous fish carcasses in inland riparian forests of the Pacific Northwest, USA. Can. J. Forest Re.s.

[27] Fox-Dobbs K, Bump JK, Peterson RO, Fox DL, Koch PL (2007). Carnivore-specific stable isotope variables and variation in the foraging ecology of modern and ancient wolf populations: case studies from Isle Royale, Minnesota, and La Brea. Can. J. Zoo.l.

[28] Payo-Payo A, Ruiz B, Cardona L, Borrell A (2013). Effect of tissue decomposition on stable isotope signatures of striped dolphins *Stenella coeruleoalba* and loggerhead sea turtles *Caretta caretta*. Aquat. Biol..

[29] Yurkowski DJ, Hussey AJ, Hussey NE, Fisk AT (2017). Effects of decomposition on carbon and nitrogen stable isotope values of muscle tissue of varying lipid content from three aquatic vertebrate species. Rapid Commun. Mass Sp..

[30] Perkins MJ (2018). Short-term tissue decomposition alters stable isotope values and C:N ratio, but does not change relationships between lipid content, C:N ratio, and Δ^13^C in marine animals. PLoS One.

[31] Burrows DG, Reichert WL, Hanson MB (2014). Effects of decomposition and storage conditions on the δ^13^C and δ^15^N isotope values of killer whale (*Orcinus orca*) skin and blubber tissues. Mar. Mammal Sci..

[32] Cobaugh KL, Schaeffer SM, DeBruyn JM (2015). Functional and structural succession of soil microbial communities below decomposing human cadavers. PLoS One.

[33] Sotiropoulos MA, Tonn WM, Wassenaar LI (2004). Effects of lipid extraction on stable carbon and nitrogen isotope analyses of fish tissues: potential consequences for food web studies. Ecol. Freshw. Fish..

[34] Hopkins JB, Whittington J, Clevenger AP, Sawaya MA, St. Clair CC (2014). Stable isotopes reveal rail-associated behavior in a threatened carnivore. Isotopes Environ. Health Stud..

[35] R Core Team. *R: A language and environment for statistical computing*, https://www.R-project.org/ (2018).

[36] Megyesi MS, Nawrocki SP, Haskell NH (2005). Using accumulated degree-days to estimate the postmortem interval from decomposed human remains. J. Forensic Sci..

[37] Bearhop S, Adams CE, Waldron S, Fuller RA, Macleod H (2004). Determining trophic niche width: a novel approach using stable isotope analysis. J. Anim. Ecol..

[38] Rubenstein DR, Hobson KA (2004). From birds to butterflies: animal movement patterns and stable isotopes. Trends Ecol. Evol..

[39] Dekeirsschieter J, Stefanuto PH, Brasseur C, Haubruge E, Focant JF (2012). Enhanced characterization of the smell of death by comprehensive two-dimensional gas chromatography-time-of-flight mass spectrometry (GCxGC-TOFMS). PLoS One.

[40] Forbes SL, Perrault KA (2014). Decomposition odour rrofiling in the air and soil surrounding vertebrate carrion. PLoS One.

[41] Carter DO, Yellowlees D, Tibbett M (2007). Cadaver decomposition in terrestrial ecosystems. Naturwissenschaften.

[42] Carter DO, Yellowlees D, Tibbett M (2008). Temperature affects microbial decomposition of cadavers (*Rattus rattus*) in contrasting soils. Appl. Soil Ecol..

[43] Roggenbuck M (2014). The microbiome of New World vultures. Nat. Commun..

[44] Mann RW, Bass WM, Meadows L (1990). Time since death and decomposition of the human body: variables and observations in case and experimental field studies. J. Forensic Sci..

