# Supplementary material for: Changes to vertebrate tissue stable isotope (δ15N) composition during decomposition
Source: Sci Rep. 2019 Jul 9;9:9929. doi: 10.1038/s41598-019-46368-5 (PMC6617491; doi:10.1038/s41598-019-46368-5)
Supplement: Supplementary file 1 — Supplementary Information [file 41598_2019_46368_MOESM1_ESM.docx]

**Supplementary Information**

**Changes to vertebrate tissue stable isotope (δ^15^N) composition during decomposition**

Sarah W. Keenan and Jennifer M. DeBruyn

**Supplemental Table S1**: Tissue types sampled and target masses (mg) for isotopic analyses.

|  | **Target Mass (mg)** | | |
| --- | --- | --- | --- |
| **Tissue Type** | **C** | **N** | **C and N** |
| Hair |  |  | 0.5 |
| Muscle |  |  | 0.5 |
| Fat | 0.5 | 5.0 |  |
| Liver |  |  | 0.5 |
| Gut tissue |  |  | 0.5 |
| Hindgut contents | 0.8 | 4.0 |  |
| Heart |  |  | 0.5 |
| Lungs |  |  | 0.5 |
| Bone |  |  | 2.0 |

**Supplementary Table S2**: Stable isotope, bulk C and N, and ratios used in this study. Data are organized according to decay stage and tissue type. N.M. = not measured.

| **Sample Name** | **Tissue type** | **Decay Stage** | **Stage** | **δ^15^N** | **%N** | **δ^13^C** | **%C** | **C/N** | **- Ln (%N)** | **- Ln (%C)** | **Δ^15^N (animal-diet)** |
| --- | --- | --- | --- | --- | --- | --- | --- | --- | --- | --- | --- |
| BND_0330_009 | bone | fresh | fresh | 6.10 | 3.73 | -26.30 | 16.07 | 4.31 | -1.32 | -2.78 | 5.54 |
| BND_0330_018 | bone | fresh | fresh | 6.52 | 3.44 | -24.53 | 11.94 | 3.47 | -1.24 | -2.48 | 5.96 |
| BND_0330_027 | bone | fresh | fresh | 1.81 | 3.59 | -24.54 | 13.92 | 3.87 | -1.28 | -2.63 | 1.26 |
| BND_1508_009 | bone | fresh | fresh | 3.75 | 3.53 | -23.61 | 12.97 | 3.67 | -1.26 | -2.56 | 3.19 |
| BND_0330_002 | fat | fresh | fresh | 4.93 | 0.72 | -33.83 | 20.23 | 28.13 | 0.33 | -3.01 | 4.38 |
| BND_0330_011 | fat | fresh | fresh | 4.75 | 0.49 | -33.33 | 19.71 | 39.92 | 0.71 | -2.98 | 4.20 |
| BND_0330_020 | fat | fresh | fresh | 1.78 | 0.78 | -34.40 | 20.18 | 25.95 | 0.25 | -3.00 | 1.22 |
| BND_1508_002 | fat | fresh | fresh | 2.01 | 0.70 | -35.71 | 72.10 | 102.82 | 0.35 | -4.28 | 1.45 |
| BND_0330_001_C | fur | fresh | fresh | 6.04 | 12.35 | -25.77 | 40.30 | 3.26 | -2.51 | -3.70 | 5.49 |
| BND_0330_010 | fur | fresh | fresh | 5.55 | 13.27 | -25.03 | 41.21 | 3.11 | -2.59 | -3.72 | 5.00 |
| BND_0330_019 | fur | fresh | fresh | 1.28 | 13.19 | -24.83 | 42.70 | 3.24 | -2.58 | -3.75 | 0.72 |
| BND_1508_001 | fur | fresh | fresh | 1.48 | 13.40 | -25.05 | 42.25 | 3.15 | -2.59 | -3.74 | 0.92 |
| BND_0330_005 | gut contents | fresh | fresh | 1.12 | 1.93 | -29.64 | 41.85 | 21.72 | -0.66 | -3.73 |  |
| BND_0330_014 | gut contents | fresh | fresh | 3.21 | 2.62 | -28.08 | 35.10 | 13.40 | -0.96 | -3.56 |  |
| BND_0330_023 | gut contents | fresh | fresh | -1.49 | 1.22 | -31.24 | 42.62 | 34.88 | -0.20 | -3.75 |  |
| BND_1508_005 | gut contents | fresh | fresh | -0.62 | 2.18 | -30.96 | 40.89 | 18.80 | -0.78 | -3.71 |  |
| BND_0330_004 | gut tissue | fresh | fresh | 3.03 | 9.41 | -26.26 | 40.64 | 4.32 | -2.24 | -3.70 | 2.48 |
| BND_0330_013 | gut tissue | fresh | fresh | 3.11 | 7.54 | -27.40 | 44.40 | 5.89 | -2.02 | -3.79 | 2.56 |
| BND_0330_022 | gut tissue | fresh | fresh | 0.44 | 9.58 | -27.89 | 45.42 | 4.74 | -2.26 | -3.82 | -0.11 |
| BND_1508_004 | gut tissue | fresh | fresh | 0.86 | 9.44 | -28.51 | 39.36 | 4.17 | -2.24 | -3.67 | 0.30 |
| BND_0330_007 | heart | fresh | fresh | 4.38 | 11.18 | -25.74 | 43.93 | 3.93 | -2.41 | -3.78 | 3.83 |
| BND_0330_016 | heart | fresh | fresh | 4.59 | 10.83 | -27.25 | 49.63 | 4.58 | -2.38 | -3.90 | 4.04 |
| BND_0330_025 | heart | fresh | fresh | 2.10 | 13.47 | -27.75 | 47.54 | 3.53 | -2.60 | -3.86 | 1.55 |
| BND_1508_007 | heart | fresh | fresh | 2.12 | 10.29 | -28.00 | 43.28 | 4.21 | -2.33 | -3.77 | 1.57 |
| BND_0330_006 | liver | fresh | fresh | 2.86 | 9.15 | -27.05 | 44.97 | 4.91 | -2.21 | -3.81 | 2.30 |
| BND_0330_015 | liver | fresh | fresh | 3.16 | 11.00 | -27.72 | 50.95 | 4.63 | -2.40 | -3.93 | 2.60 |
| BND_0330_024 | liver | fresh | fresh | 1.07 | 10.01 | -28.69 | 49.23 | 4.92 | -2.30 | -3.90 | 0.52 |
| BND_1508_008 | liver | fresh | fresh | 1.03 | 10.50 | -29.45 | 47.35 | 4.51 | -2.35 | -3.86 | 0.47 |
| BND_0330_008 | lungs | fresh | fresh | 3.59 | 11.79 | -25.97 | 41.56 | 3.52 | -2.47 | -3.73 | 3.04 |
| BND_0330_017 | lungs | fresh | fresh | 3.57 | 12.28 | -25.61 | 46.83 | 3.81 | -2.51 | -3.85 | 3.02 |
| BND_0330_026 | lungs | fresh | fresh | 0.95 | 12.28 | -27.33 | 46.76 | 3.81 | -2.51 | -3.85 | 0.40 |
| BND_1508_006 | lungs | fresh | fresh | 1.13 | 13.07 | -27.66 | 46.84 | 3.58 | -2.57 | -3.85 | 0.58 |
| BND_0330_003 | muscle | fresh | fresh | 4.25 | 12.08 | -25.54 | 42.66 | 3.53 | -2.49 | -3.75 | 3.69 |
| BND_0330_012 | muscle | fresh | fresh | 4.35 | 14.96 | -25.52 | 44.42 | 2.97 | -2.71 | -3.79 | 3.80 |
| BND_0330_021 | muscle | fresh | fresh | 0.57 | 12.94 | -25.43 | 44.25 | 3.42 | -2.56 | -3.79 | 0.02 |
| BND_1508_003 | muscle | fresh | fresh | 1.49 | 12.77 | -27.19 | 43.74 | 3.43 | -2.55 | -3.78 | 0.94 |
| BND_0404_009 | bone | early bloat | bloat | 4.43 | 4.05 | -23.81 | 14.19 | 3.50 | -1.40 | -2.65 | 3.87 |
| BND_0404_018 | bone | early bloat | bloat | 4.69 | 3.70 | -22.24 | 14.32 | 3.87 | -1.31 | -2.66 | 4.13 |

**Supplementary Table S2** (continued)

| **Sample Name** | **Tissue type** | **Decay Stage** | **Stage** | **δ^15^N** | **%N** | **δ^13^C** | **%C** | **C/N** | **- Ln (%N)** | **- Ln (%C)** | **Δ^15^N (animal-diet)** |
| --- | --- | --- | --- | --- | --- | --- | --- | --- | --- | --- | --- |
| BND_0404_027 | bone | early bloat | bloat | 4.05 | 3.97 | -24.75 | 13.38 | 3.37 | -1.38 | -2.59 | 3.50 |
| BND_0407_009 | bone | late bloat | bloat | 3.63 | 3.63 | -23.12 | 13.24 | 3.65 | -1.29 | -2.58 | 3.08 |
| BND_0407_018 | bone | late bloat | bloat | 5.28 | 3.73 | -24.96 | 14.97 | 4.01 | -1.32 | -2.71 | 4.72 |
| BND_0407_027 | bone | late bloat | bloat | 4.11 | 3.48 | -19.70 | 11.87 | 3.41 | -1.25 | -2.47 | 3.56 |
| BND_0404_002 | fat | early bloat | bloat | 3.72 | 0.76 | -35.89 | 68.59 | 89.98 | 0.27 | -4.23 | 3.17 |
| BND_0404_011 | fat | early bloat | bloat | 1.75 | 0.32 | -32.27 | 20.02 | 62.84 | 1.14 | -3.00 | 1.20 |
| BND_0404_020 | fat | early bloat | bloat | 3.38 | 0.52 | -33.32 | 55.41 | 107.58 | 0.66 | -4.01 | 2.83 |
| BND_0407_002 | fat | late bloat | bloat | 3.61 | 0.82 | -33.47 | 19.87 | 24.09 | 0.19 | -2.99 | 3.05 |
| BND_0407_011 | fat | late bloat | bloat | 3.86 | 0.96 | -34.00 | 19.29 | 20.09 | 0.04 | -2.96 | 3.31 |
| BND_0407_020 | fat | late bloat | bloat | 4.51 | 0.83 | -30.91 | 19.71 | 23.63 | 0.18 | -2.98 | 3.95 |
| BND_0404_001_C | fur | early bloat | bloat | 4.86 | 13.67 | -23.21 | 42.56 | 3.11 | -2.62 | -3.75 | 4.30 |
| BND_0404_010_C | fur | early bloat | bloat | 4.72 | 13.71 | -22.04 | 43.56 | 3.18 | -2.62 | -3.77 | 4.17 |
| BND_0404_019 | fur | early bloat | bloat | 4.62 | 12.74 | -24.91 | 40.90 | 3.21 | -2.54 | -3.71 | 4.07 |
| BND_0407_005_C | fur | late bloat | bloat | 3.62 | 14.40 | -24.60 | 42.81 | 2.97 | -2.67 | -3.76 | 3.06 |
| BND_0407_014 | fur | late bloat | bloat | 3.77 | 14.96 | -27.90 | 46.61 | 3.12 | -2.71 | -3.84 | 3.21 |
| BND_0407_023 | fur | late bloat | bloat | 4.73 | 13.31 | -21.90 | 43.19 | 3.25 | -2.59 | -3.77 | 4.17 |
| BND_0404_005 | gut contents | early bloat | bloat | 3.14 | 2.68 | -29.64 | 38.92 | 14.50 | -0.99 | -3.66 | 2.59 |
| BND_0404_014 | gut contents | early bloat | bloat | 1.30 | 1.55 | -26.95 | 42.98 | 27.65 | -0.44 | -3.76 | 0.74 |
| BND_0404_023 | gut contents | early bloat | bloat | 2.07 | 3.26 | -29.00 | 39.19 | 12.01 | -1.18 | -3.67 | 1.51 |
| BND_0407_004 | gut contents | late bloat | bloat | 2.92 | 3.31 | -28.72 | 42.00 | 12.68 | -1.20 | -3.74 | 2.36 |
| BND_0407_013 | gut contents | late bloat | bloat | 2.05 | 3.13 | -31.44 | 40.21 | 12.86 | -1.14 | -3.69 | 1.50 |
| BND_0407_022 | gut contents | late bloat | bloat | 5.35 | 3.43 | -30.51 | 40.61 | 11.84 | -1.23 | -3.70 | 4.79 |
| BND_0404_004 | gut tissue | early bloat | bloat | 3.42 | 9.39 | -26.49 | 45.85 | 4.88 | -2.24 | -3.83 | 2.87 |
| BND_0404_013 | gut tissue | early bloat | bloat | 2.75 | 11.87 | -25.00 | 45.12 | 3.80 | -2.47 | -3.81 | 2.20 |
| BND_0404_022 | gut tissue | early bloat | bloat | 2.56 | 7.93 | -27.42 | 38.64 | 4.87 | -2.07 | -3.65 | 2.00 |
| BND_0407_003 | gut tissue | late bloat | bloat | 3.34 | 9.91 | -27.31 | 47.88 | 4.83 | -2.29 | -3.87 | 2.79 |
| BND_0407_012 | gut tissue | late bloat | bloat | 3.33 | 10.31 | -29.23 | 46.54 | 4.51 | -2.33 | -3.84 | 2.78 |
| BND_0407_021 | gut tissue | late bloat | bloat | 5.14 | 10.70 | -25.88 | 46.53 | 4.35 | -2.37 | -3.84 | 4.59 |
| BND_0404_007 | heart | early bloat | bloat | 5.08 | 12.15 | -26.14 | 48.27 | 3.97 | -2.50 | -3.88 | 4.52 |
| BND_0404_016 | heart | early bloat | bloat | 4.01 | 12.92 | -24.94 | 47.97 | 3.71 | -2.56 | -3.87 | 3.45 |
| BND_0404_025 | heart | early bloat | bloat | 3.93 | 12.35 | -26.56 | 46.81 | 3.79 | -2.51 | -3.85 | 3.38 |
| BND_0407_007 | heart | late bloat | bloat | 3.96 | 12.33 | -26.73 | 48.89 | 3.96 | -2.51 | -3.89 | 3.41 |
| BND_0407_016 | heart | late bloat | bloat | 4.55 | 12.05 | -29.06 | 49.78 | 4.13 | -2.49 | -3.91 | 4.00 |
| BND_0407_025 | heart | late bloat | bloat | 5.94 | 12.36 | -24.34 | 47.21 | 3.82 | -2.51 | -3.85 | 5.39 |
| BND_0404_006 | liver | early bloat | bloat | 4.12 | 9.88 | -28.48 | 50.91 | 5.15 | -2.29 | -3.93 | 3.57 |
| BND_0404_015 | liver | early bloat | bloat | 3.08 | 12.03 | -26.15 | 49.83 | 4.14 | -2.49 | -3.91 | 2.53 |
| BND_0404_024 | liver | early bloat | bloat | 2.63 | 8.95 | -29.04 | 52.45 | 5.86 | -2.19 | -3.96 | 2.07 |
| BND_0407_006 | liver | late bloat | bloat | 2.87 | 6.88 | -30.41 | 56.68 | 8.24 | -1.93 | -4.04 | 2.32 |
| BND_0407_015 | liver | late bloat | bloat | 3.79 | 10.22 | -30.43 | 50.26 | 4.92 | -2.32 | -3.92 | 3.24 |

**Supplementary Table S2** (continued)

| **Sample Name** | **Tissue type** | **Decay Stage** | **Stage** | **δ^15^N** | **%N** | **δ^13^C** | **%C** | **C/N** | **- Ln (%N)** | **- Ln (%C)** | **Δ^15^N (animal-diet)** |
| --- | --- | --- | --- | --- | --- | --- | --- | --- | --- | --- | --- |
| BND_0407_024 | liver | late bloat | bloat | 6.00 | 9.45 | -26.36 | 49.68 | 5.26 | -2.25 | -3.91 | 5.44 |
| BND_0404_008 | lungs | early bloat | bloat | 4.08 | 13.00 | -26.41 | 46.26 | 3.56 | -2.57 | -3.83 | 3.52 |
| BND_0404_017 | lungs | early bloat | bloat | 2.99 | 13.42 | -25.17 | 51.21 | 3.82 | -2.60 | -3.94 | 2.44 |
| BND_0404_026 | lungs | early bloat | bloat | 2.62 | 14.49 | -26.64 | 46.03 | 3.18 | -2.67 | -3.83 | 2.07 |
| BND_0407_008 | lungs | late bloat | bloat | 3.45 | 12.16 | -26.06 | 45.01 | 3.70 | -2.50 | -3.81 | 2.89 |
| BND_0407_017 | lungs | late bloat | bloat | 4.01 | 12.13 | -28.42 | 47.14 | 3.89 | -2.50 | -3.85 | 3.46 |
| BND_0407_026 | lungs | late bloat | bloat | 4.95 | 11.69 | -23.99 | 45.04 | 3.85 | -2.46 | -3.81 | 4.40 |
| BND_0407_028 | maggots | late bloat | bloat | 6.94 | 9.62 | -25.75 | 42.84 | 4.45 | -2.26 | -3.76 | 6.38 |
| BND_0404_003 | muscle | early bloat | bloat | 3.81 | 13.61 | -24.94 | 44.53 | 3.27 | -2.61 | -3.80 | 3.26 |
| BND_0404_012 | muscle | early bloat | bloat | 3.88 | 13.67 | -23.94 | 45.97 | 3.36 | -2.62 | -3.83 | 3.32 |
| BND_0404_021 | muscle | early bloat | bloat | 3.52 | 13.17 | -26.30 | 44.82 | 3.40 | -2.58 | -3.80 | 2.97 |
| BND_0407_001 | muscle | late bloat | bloat | 4.04 | 13.97 | -25.88 | 45.81 | 3.28 | -2.64 | -3.82 | 3.49 |
| BND_0407_010 | muscle | late bloat | bloat | 4.22 | 13.18 | -27.97 | 46.55 | 3.53 | -2.58 | -3.84 | 3.66 |
| BND_0407_019 | muscle | late bloat | bloat | 5.28 | 12.32 | -23.31 | 44.91 | 3.65 | -2.51 | -3.80 | 4.73 |
| BND_0414_009 | bone | early active | active | 8.25 | 3.60 | -23.19 | 12.40 | 3.44 | -1.28 | -2.52 | 7.70 |
| BND_0414_019 | bone | early active | active | 6.49 | 3.79 | -20.42 | 13.11 | 3.46 | -1.33 | -2.57 | 5.93 |
| BND_0414_029 | bone | early active | active | 4.88 | 3.62 | -19.42 | 13.50 | 3.73 | -1.29 | -2.60 | 4.33 |
| BND_0417_006 | bone | late active | active | 5.05 | 3.94 | -20.76 | 13.53 | 3.43 | -1.37 | -2.60 | 4.49 |
| BND_0417_018 | bone | late active | active | 5.76 | 5.23 | -24.46 | 18.35 | 3.51 | -1.65 | -2.91 | 5.20 |
| BND_0417_022 | bone | late active | active | 4.83 | 3.69 | -21.77 | 12.87 | 3.48 | -1.31 | -2.55 | 4.27 |
| BND_0414_002 | fat | early active | active | 6.96 | 0.92 | -35.24 | 19.75 | 21.39 | 0.08 | -2.98 | 6.41 |
| BND_0414_012 | fat | early active | active | 6.14 | 1.78 | -31.23 | 19.21 | 10.76 | -0.58 | -2.96 | 5.58 |
| BND_0414_022 | fat | early active | active | 3.80 | 0.84 | -31.36 | 19.91 | 23.76 | 0.18 | -2.99 | 3.25 |
| BND_0417_004 | fat | late active | active | 6.05 | 1.77 | -30.78 | 19.29 | 10.91 | -0.57 | -2.96 | 5.49 |
| BND_0417_012 | fat | late active | active | 7.29 | 0.77 | -31.88 | 19.82 | 25.60 | 0.26 | -2.99 | 6.73 |
| BND_0417_020 | fat | late active | active | 4.64 | 1.00 | -31.39 | 19.58 | 19.54 | 0.00 | -2.97 | 4.08 |
| BND_0414_003_C | fur | early active | active | 5.89 | 13.76 | -23.50 | 43.46 | 3.16 | -2.62 | -3.77 | 5.33 |
| BND_0414_013 | fur | early active | active | 5.52 | 14.22 | -23.66 | 42.94 | 3.02 | -2.65 | -3.76 | 4.96 |
| BND_0414_023_C | fur | early active | active | 5.61 | 14.79 | -21.75 | 45.35 | 3.07 | -2.69 | -3.81 | 5.06 |
| BND_0417_005_C | fur | late active | active | 5.70 | 11.14 | -23.48 | 44.36 | 3.98 | -2.41 | -3.79 | 5.15 |
| BND_0417_013 | fur | late active | active | 5.82 | 14.26 | -24.32 | 45.49 | 3.19 | -2.66 | -3.82 | 5.26 |
| BND_0417_021_C | fur | late active | active | 6.06 | 12.84 | -22.86 | 43.11 | 3.36 | -2.55 | -3.76 | 5.51 |
| BND_0414_005 | gut contents | early active | active | 4.52 | 2.22 | N.M. | N.M. |  | -0.80 |  | 3.97 |
| BND_0414_015 | gut contents | early active | active | 3.04 | 2.12 | N.M. | N.M. |  | -0.75 |  | 2.49 |
| BND_0414_025 | gut contents | early active | active | 3.28 | 3.78 | -28.76 | 41.31 | 10.94 | -1.33 | -3.72 | 2.72 |
| BND_0417_008 | gut contents | late active | active | 4.07 | 3.78 | -27.55 | 41.01 | 10.85 | -1.33 | -3.71 | 3.52 |
| BND_0417_016 | gut contents | late active | active | 5.24 | 2.89 | -29.82 | 39.66 | 13.71 | -1.06 | -3.68 | 4.69 |
| BND_0417_025 | gut contents | late active | active | 9.98 | 1.34 | -28.95 | 42.72 | 31.88 | -0.29 | -3.75 | 9.43 |
| BND_0414_004 | gut tissue | early active | active | 4.69 | 10.70 | -26.33 | 50.50 | 4.72 | -2.37 | -3.92 | 4.14 |

**Supplementary Table S2** (continued)

| **Sample Name** | **Tissue type** | **Decay Stage** | **Stage** | **δ^15^N** | **%N** | **δ^13^C** | **%C** | **C/N** | **- Ln (%N)** | **- Ln (%C)** | **Δ^15^N (animal-diet)** |
| --- | --- | --- | --- | --- | --- | --- | --- | --- | --- | --- | --- |
| BND_0414_014 | gut tissue | early active | active | 6.32 | 8.52 | -28.32 | 46.81 | 5.49 | -2.14 | -3.85 | 5.77 |
| BND_0414_024 | gut tissue | early active | active | 3.19 | 6.12 | -27.53 | 45.69 | 7.47 | -1.81 | -3.82 | 2.64 |
| BND_0417_007 | gut tissue | late active | active | 3.95 | 7.52 | -27.25 | 51.76 | 6.88 | -2.02 | -3.95 | 3.40 |
| BND_0417_015 | gut tissue | late active | active | 6.41 | 8.13 | -28.82 | 48.84 | 6.01 | -2.10 | -3.89 | 5.86 |
| BND_0417_024 | gut tissue | late active | active | 6.09 | 8.15 | -27.29 | 47.70 | 5.85 | -2.10 | -3.86 | 5.54 |
| BND_0414_007 | heart | early active | active | 7.74 | 12.53 | -25.89 | 49.10 | 3.92 | -2.53 | -3.89 | 7.18 |
| BND_0414_017 | heart | early active | active | 5.98 | 12.17 | -26.53 | 48.86 | 4.01 | -2.50 | -3.89 | 5.43 |
| BND_0414_027 | heart | early active | active | 4.96 | 11.02 | -24.19 | 46.69 | 4.24 | -2.40 | -3.84 | 4.41 |
| BND_0414_008 | liver | early active | active | 5.12 | 9.57 | -27.37 | 51.60 | 5.39 | -2.26 | -3.94 | 4.57 |
| BND_0414_018 | liver | early active | active | 5.46 | 9.10 | -28.82 | 53.84 | 5.92 | -2.21 | -3.99 | 4.90 |
| BND_0414_028 | liver | early active | active | 4.08 | 7.09 | -26.68 | 53.37 | 7.53 | -1.96 | -3.98 | 3.52 |
| BND_0417_009 | liver | late active | active | 5.05 | 9.08 | -27.15 | 58.88 | 6.48 | -2.21 | -4.08 | 4.50 |
| BND_0417_017 | liver | late active | active | 7.16 | 7.28 | -30.92 | 56.82 | 7.81 | -1.99 | -4.04 | 6.60 |
| BND_0414_020 | lungs | early active | active | 5.97 | 10.13 | -27.75 | 49.86 | 4.92 | -2.32 | -3.91 | 5.41 |
| BND_0414_030 | lungs | early active | active | 4.56 | 9.31 | -24.75 | 46.84 | 5.03 | -2.23 | -3.85 | 4.00 |
| BND_0414_006 | maggots | early active | active | 8.01 | 10.30 | -26.18 | 46.78 | 4.54 | -2.33 | -3.85 | 7.46 |
| BND_0414_010 | maggots | early active | active | 7.57 | 7.82 | -26.45 | 45.09 | 5.76 | -2.06 | -3.81 | 7.02 |
| BND_0414_016 | maggots | early active | active | 7.60 | 9.09 | -24.56 | 44.26 | 4.87 | -2.21 | -3.79 | 7.04 |
| BND_0414_026 | maggots | early active | active | 7.67 | 8.58 | -24.94 | 44.33 | 5.16 | -2.15 | -3.79 | 7.12 |
| BND_0417_001 | maggots | late active | active | 7.84 | 6.79 | -28.73 | 50.67 | 7.47 | -1.92 | -3.93 | 7.29 |
| BND_0417_002 | maggots | late active | active | 6.51 | 7.49 | -27.25 | 52.06 | 6.95 | -2.01 | -3.95 | 5.95 |
| BND_0417_010 | maggots | late active | active | 6.56 | 8.07 | -25.28 | 48.77 | 6.05 | -2.09 | -3.89 | 6.00 |
| BND_0417_014 | maggots | late active | active | 7.27 | 7.97 | -27.34 | 49.97 | 6.27 | -2.08 | -3.91 | 6.72 |
| BND_0417_023 | maggots | late active | active | 7.26 | 7.86 | -27.69 | 50.24 | 6.39 | -2.06 | -3.92 | 6.70 |
| BND_0414_001 | muscle | early active | active | 5.70 | 13.38 | -25.33 | 46.59 | 3.48 | -2.59 | -3.84 | 5.15 |
| BND_0414_011 | muscle | early active | active | 5.94 | 12.96 | -25.55 | 46.39 | 3.58 | -2.56 | -3.84 | 5.38 |
| BND_0414_021 | muscle | early active | active | 5.04 | 12.55 | -24.44 | 46.37 | 3.69 | -2.53 | -3.84 | 4.49 |
| BND_0417_003 | muscle | late active | active | 5.97 | 12.76 | -23.28 | 46.52 | 3.65 | -2.55 | -3.84 | 5.41 |
| BND_0417_011 | muscle | late active | active | 6.88 | 12.56 | -26.74 | 47.26 | 3.76 | -2.53 | -3.86 | 6.32 |
| BND_0417_019 | muscle | late active | active | 4.75 | 13.33 | -24.61 | 46.84 | 3.51 | -2.59 | -3.85 | 4.20 |

**Supplementary Table S3**: Result of one-way ANOVA tests for significant differences in tissue composition between decomposition stages. Each one-way ANOVA was conducted by evaluating the differences within each measured parameter (i.e., C/N) from tissues collected during fresh, bloat, and active stages. Compositional changes that were significant at α < 0.05 are in bold. Full datasets are presented in Table 1 and Supplementary Table S2.

|  | **δ^15^N** | | **%N** | | **δ^13^C** | | **%C** | | **C/N** | | **Δ^15^N_animal-diet_** | |
| --- | --- | --- | --- | --- | --- | --- | --- | --- | --- | --- | --- | --- |
|  | p | F | p | F | p | F | p | F | p | F | p | F |
| Bone | 0.17 | 2.05 | 0.34 | 1.19 | 0.054 | 3.68 | 0.95 | 0.05 | 0.17 | 2.08 | 0.17 | 2.05 |
| Fat | **0.012** | 6.26 | **0.04** | 4.15 | 0.09 | 2.92 |  |  |  |  | **0.01** | 6.26 |
| Hair | 0.051 | 3.77 | 0.52 | 0.69 | 0.19 | 1.93 | 0.06 | 3.59 | 0.51 | 0.71 | 0.052 | 3.76 |
| Gut tissue | **0.004** | 8.66 | 0.09 | 2.89 | 0.54 | 0.65 | **0.016** | 5.73 | **0.01** | 6.79 | **0.004** | 8.65 |
| Heart | **0.022** | 5.72 | 0.30 | 1.35 | 0.34 | 1.22 | 0.24 | 1.63 | 0.59 | 0.56 | **0.022** | 5.72 |
| Liver | **0.004** | 9.02 | 0.17 | 2.03 | 0.95 | 0.05 | **0.01** | 6.88 | 0.07 | 3.36 | **0.004** | 9.04 |
| Lungs | **0.036** | 4.93 | **0.005** | 10.09 | 0.85 | 0.16 | 0.42 | 0.97 | **<0.001** | 29.59 | **0.036** | 4.92 |
| Muscle | **0.003** | 9.36 | 0.65 | 0.45 | 0.58 | 0.58 | **<0.001** | 23.37 | **0.049** | 3.85 | **0.003** | 9.37 |
| Gut contents | **0.017** | 5.68 | 0.23 | 1.63 | 0.49 | 0.77 | 0.79 | 0.24 | 0.44 | 0.88 |  |  |
